# Cardiovascular Health in Pregnant Women and Their Off-Spring

**DOI:** 10.3390/jcm12093293

**Published:** 2023-05-05

**Authors:** Gizem Seyda Erbas, Felicia Nordenstam, Renate Oberhoffer-Fritz, Christina Sitzberger, Annette Wacker-Gußmann

**Affiliations:** 1 Department of Congenital Heart Disease and Pediatric Cardiology, German Heart Center, 80636 Munich, Germanyannette.wacker-gussmann@tum.de (A.W.-G.); 2 Department of Pediatric Cardiology, Department of Woman’s and Children’s Health Karolinska Institute, Karolinska University Hospital, 17176 Stockholm, Sweden; 3 Institute of Preventive Pediatrics, Faculty of Sport and Health Sciences, Technical University of Munich, 80992 Munich, Germany

Child development requires complex interactions between the pregnant woman and the growing child. Different risk factors of the pregnant woman, such as overweight and obesity, hypertension, and diabetes, as well as pregnancy-associated factors such as reproductive technologies, are of crucial importance. Over the life course, a number of behavioral, environmental, and psychosocial risk factors also contribute to cardiovascular development. In this Special Issue, a number of different articles highlight critical elements of cardiovascular risk factors in pregnancy and their impact on fetal development, as well as address the short- and long-term health of both the mother and child.

Obesity is a world-wide growing problem, which impacts obstetrical care. In pregnancy with overweight/obese women, the risk of complications at birth and cardiovascular diseases, such as hypertension and gestational diabetes in the long-term, are increased [1]. However, there is not only an impact on the pregnant women herself; there is also an impact on the child. Children of obese mothers are more likely to have structural or valvular heart defects and diastolic heart failure [2]. Changes at the cellular level, that promote atherosclerosis and thus contribute to increased cardiovascular risk, have been observed in children exposed to maternal diabetes in utero [3]. Hypertension in pregnancy can also influence cardiovascular diseases in infants. These include the more frequent occurrence of hypertension in the children themselves, heart failure, and cardiac arrhythmias [4].

In this Issue, we also focus on acquired issues, such as children conceived by assisted reproductive technologies (ARTs). A comparison of children conceived by ART with children conceived naturally shows that ART children have significantly lower myocardial velocity in early diastole and increased left ventricular filling pressure from the age of 10 years, and thus ART also represents a risk factor for cardiovascular disease [5].

In conclusion, pregnancy represents a particularly important period for the maintenance of cardiovascular health in both mother and child. Control of the cardiovascular risk factors and targeted care for those that are affected is essential for improved maternal and infant outcome and cardiovascular health. Many more topics than those that are addressed in this Issue must be addressed in the near future to prevent cardiovascular complications in the next generation, thus ensuring that the healthcare system remains affordable.
